# Genomic Biomarkers for First-Line Treatment Selection in Metastatic Pancreatic Ductal Adenocarcinoma: A Narrative Review

**DOI:** 10.3390/cancers18101664

**Published:** 2026-05-21

**Authors:** Anushareddy Muddasani, Ahmed Abdelnoor, Ashish Manne

**Affiliations:** 1 Department of Internal Medicine, University of Arkansas for Medical Sciences, Little Rock, AR 72205, USA; 2 Department of Internal Medicine, Zagazig University, Zazazig 44519, Egypt; ahmedtamer137@gmail.com; 3 Department of Internal Medicine, Division of Medical Oncology, The Ohio State University Wexner Medical Center, Columbus, OH 43210, USA; ashish.manne@osumc.edu

**Keywords:** pancreatic ductal adenocarcinoma, metastatic pancreatic cancer, predictive biomarkers, FOLFIRINOX, gemcitabine plus nab-paclitaxel, homologous recombination deficiency, transcriptomic subtypes, precision oncology, biomarker-guided therapy

## Abstract

Metastatic pancreatic ductal adenocarcinoma (PDAC) is typically treated with fluorouracil, leucovorin, irinotecan, and oxaliplatin (FOLFIRINOX) or gemcitabine plus nab-paclitaxel (GnP), but the choice between regimens remains largely empirical. This narrative review summarizes biomarkers with potential to inform first-line selection in metastatic PDAC, emphasizing genomic and transcriptomic correlates of differential benefit. Recent head-to-head trials, particularly Pancreatic Adenocarcinoma Signature Stratification for Treatment (PASS-01) and GENERATE (Japan Clinical Oncology Group [JCOG] 1611), indicate that modified FOLFIRINOX (mFOLFIRINOX) is not uniformly superior to GnP, strengthening the rationale for biomarker-guided selection. The strongest evidence favoring platinum-based/FOLFIRINOX strategies involves homologous recombination repair deficiency (HRD), especially alterations in germline breast cancer gene 1/2 (*BRCA1/2*) or partner and localizer of BRCA2 (*PALB2*), as well as broader genomic scar signatures. Transcriptomic subtype and GATA-binding protein 6 (*GATA6*) expression are promising but remain unsettled because retrospective data favor classical/*GATA6*-high disease for FOLFIRINOX, whereas PASS-01 suggested better outcomes with GnP in classical tumors. Candidate biomarkers favoring GnP include high human equilibrative nucleoside transporter 1 (hENT1), low class III β-tubulin (*TUBB3)* expression, and exploratory phosphatidylinositol 3-kinase (*PI3K*)/*KIT/NOTCH* pathway mutation signals. Comprehensive molecular profiling also identifies actionable alterations that may redirect patients to targeted therapy or clinical trials rather than standard chemotherapy alone. Importantly, no biomarker has yet been prospectively validated in a biomarker-stratified randomized trial with regimen selection as the primary endpoint; all biomarker-regimen associations described in this review should therefore be considered hypothesis-generating rather than practice-defining. Nevertheless, the convergence of genomic, transcriptomic, and organoid-based approaches makes biologically informed first-line selection increasingly feasible in metastatic PDAC.

## Simple Summary

Patients with metastatic pancreatic ductal adenocarcinoma are typically treated with one of two first-line chemotherapy combinations, but clinicians currently lack reliable tools to determine which regimen will work best for an individual patient. This gap has become more clinically urgent following recent randomized trials showing that neither regimen is clearly superior in unselected populations. This review evaluates the growing body of evidence supporting tumor-based molecular markers as potential tools for matching patients to the most effective chemotherapy. By identifying which biological features of a tumor predict benefit from one regimen over the other, this work aims to support the transition from empirical treatment selection toward a precision oncology approach in a disease where even modest improvements in treatment matching could meaningfully impact patient outcomes.

## 1. Introduction

Pancreatic ductal adenocarcinoma (PDAC) is characterized by aggressive biology, late presentation, and poor long-term survival. In routine clinical practice, most patients with PDAC present with unresectable or metastatic disease, making systemic therapy the principal treatment modality. Although advances in supportive care and regimen intensification have modestly improved outcomes, metastatic PDAC remains a disease in which median survival is commonly measured in months rather than years [1].

PDAC is the most common histologic subtype of pancreatic cancer, accounting for approximately 90% of cases, with roughly 60,000 new diagnoses annually in the United States and an incidence rising by 0.5–1% per year. The disease is projected to become the second-leading cause of cancer-related mortality by 2030. The median age at diagnosis is 71 years, with a slight male predominance (male-to-female ratio approximately 1.3:1) [2].

Modifiable risk factors include tobacco smoking (twofold increased risk), chronic pancreatitis, obesity, and diabetes mellitus, while non-modifiable factors include age, sex, and germline predisposition syndromes involving breast cancer gene 1/2 *(BRCA1/2*), partner and localizer of BRCA2 *(PALB2*), ataxia-telangiectasia mutated (*ATM*), cyclin-dependent kinase inhibitor 2A (*CDKN2A*), and mismatch repair (*MMR*) genes. Approximately 50–57% of patients present with metastatic disease at diagnosis, 30–35% with locally advanced unresectable disease, and only 10–20% with potentially resectable tumors. The five-year overall survival (OS) for metastatic PDAC remains approximately 3%, with a median OS of less than one year with current standard regimens [1,2,3].

Fluorouracil, leucovorin, irinotecan, and oxaliplatin (FOLFIRINOX) and gemcitabine plus nab-paclitaxel (GnP) are the two most widely used first-line regimens for fit patients with metastatic PDAC. In the PRODIGE/ACCORD trial, FOLFIRINOX improved median OS (11.1 vs. 6.8 months), progression-free survival (PFS), and response rate over gemcitabine monotherapy [4]. In contemporary practice, modified FOLFIRINOX (mFOLFIRINOX) with reduced irinotecan dosing and elimination of the fluorouracil bolus is the regimen most commonly used, offering a more favorable toxicity profile while maintaining efficacy. In the MPACT trial, GnP also improved survival outcomes relative to gemcitabine alone (median OS 8.5 vs. 6.7 months) and established itself as an important alternative for patients in whom a gemcitabine-based approach is preferred [5].

More recently, the NAPOLI-3 trial introduced NALIRIFOX (liposomal irinotecan, fluorouracil, leucovorin, oxaliplatin), demonstrating a statistically significant improvement in OS (11.1 vs. 9.2 months; hazard ratio (HR) 0.83; *p* = 0.036) and PFS (7.4 vs. 5.6 months; HR 0.69; *p* =0.0001) compared with GnP [6]. However, NALIRIFOX has not been directly compared with FOLFIRINOX or mFOLFIRINOX in randomized trials, and its role in the treatment algorithm relative to these regimens remains undefined. Despite these advances, all three regimens were developed and tested in molecularly unselected populations.

In the absence of validated predictive biomarkers, clinicians still choose between these regimens largely based on performance status, age, neuropathy risk, biliary stenting status, patient preference, and anticipated toxicity.

Emerging evidence, however, suggests that PDAC is not a single disease but encompasses biologically distinct subgroups with differing therapeutic vulnerabilities. Up to 20–25% of PDACs harbor actionable molecular alterations, most commonly in DNA damage response (DDR) pathways, MMR, and rare oncogenic fusions. The Know Your Tumor registry trial demonstrated that patients with actionable alterations who received molecularly matched therapy experienced a median OS approximately one year longer than those receiving unmatched therapy (2.58 vs. 1.51), a magnitude of benefit unmatched by any other therapeutic modality in this population [7].

Programs such as the Canadian Oncology Molecular Profiling in Advanced Cancers Trial (COMPASS) demonstrated that PDAC contains biologically distinct subgroups differing in transcriptomic phenotype, genomic architecture, DNA repair status, inflammatory status, and clinical outcomes, including survival and treatment response [8].

The recent publication of head-to-head randomized trials, Pancreatic Adenocarcinoma Signature Stratification for Treatment (PASS-01) and GENERATE (Japan Clinical Oncology Group [JCOG] 1611), has further transformed the landscape. In PASS-01, a phase II trial, median OS was 8.5 months with mFOLFIRINOX versus 9.7 months with GnP (HR 1.57; *p* = 0.017), favoring GnP [9]. In GENERATE (JCOG1611), a phase II/III trial that stopped early for futility, median OS was 14.0 months with mFOLFIRINOX versus 17.1 months with GnP (HR 1.31; 95% confidence interval (CI) 0.97–1.77) [10]. These findings underscore the limitations of a one-size-fits-all approach and highlight the need for biologically informed treatment selection. These randomized studies suggest that mFOLFIRINOX may not be universally superior to GnP in unselected populations, reinforcing the need for biologically informed treatment selection. However, implementing precision oncology in PDAC faces substantial practical challenges, including limited tissue availability from small-gauge biopsies, prolonged turnaround times for comprehensive molecular profiling that may delay treatment initiation, and the absence of prospectively validated biomarker-regimen algorithms. These barriers underscore the gap between the biological rationale for biomarker-guided selection and its routine clinical application.

The goal of this review is therefore not to catalog all molecular abnormalities in PDAC, but to focus on those biomarkers most relevant to first-line treatment selection between mFOLFIRINOX and GnP in the metastatic setting. We also briefly discuss actionable alterations that may redirect patients toward targeted therapy or clinical trials, as this information is inseparable from contemporary treatment planning.

## 2. Literature Search Strategy

A literature search was performed to identify studies evaluating biomarkers that may guide first-line treatment selection between FOLFIRINOX and GnP in metastatic PDAC. Electronic databases, including PubMed, Scopus, and Web of Science, were searched from inception through April 2026 using combinations of the following terms: “pancreatic ductal adenocarcinoma”, “metastatic pancreatic cancer”, “FOLFIRINOX”, “gemcitabine nab-paclitaxel”, “biomarker”, “predictive marker”, “homologous recombination deficiency”, “BRCA”, “transcriptomic subtype”, “KRAS”, “hENT1”, “GATA6”, “molecular profiling”, and “precision oncology”. Since this is a narrative review, study selection was based on clinical relevance and scientific quality rather than a formal systematic screening protocol with predefined inclusion/exclusion checklists or PRISMA-compliant methodology. Reference lists of included articles and recent review articles were manually screened to identify additional relevant studies. Only peer-reviewed human studies published in English were included. Eligible studies comprised landmark phase III chemotherapy trials, prospective molecular profiling cohorts, translational biomarker studies, molecularly annotated retrospective series, meta-analyses, and recent review articles directly relevant to biomarker-guided regimen selection in advanced PDAC. Published conference abstracts from major oncology meetings were included when they provided unique biomarker-regimen data. Editorials, commentaries, animal-only studies, and case reports without biomarker or molecular profiling data were excluded. Articles were screened by title and abstract for relevance to biomarker-guided regimen selection, and full texts were reviewed for those meeting the above criteria; final inclusion was determined by consensus among the authors. Because several clinically informative biomarker studies enrolled mixed unresectable stage III/IV populations rather than exclusively metastatic cohorts, those studies were included when they provided treatment-specific molecular insights. However, their results are interpreted cautiously and framed in relation to metastatic practice.

## 3. Head-to-Head Trial Evidence: Why Biomarker Selection Matters

Understanding the comparative efficacy of FOLFIRINOX and GnP provides essential context for biomarker-guided selection. Historically, the preference for FOLFIRINOX was based on cross-trial comparison: in the PRODIGE/ACCORD trial, FOLFIRINOX produced a median OS of 11.1 months, an objective response rate (ORR) of 31.6%, and a median PFS of 6.4 months [4], whereas in the MPACT trial, GnP achieved a median OS of 8.5 months, an ORR of 23%, and a median PFS of 5.5 months [5]. However, cross-trial comparisons are inherently unreliable because the PRODIGE trial enrolled younger, fitter patients (median age 61 years, restricted to Eastern Cooperative Oncology Group (ECOG) performance status (PS) 0–1), while the MPACT trial included older patients (median age 63 years) and permitted ECOG PS 2 [4,5]. This guideline-level equipoise is now reflected in the National Comprehensive Cancer Network (NCCN) guidelines (v1.2026), which list albumin-bound paclitaxel/gemcitabine, FOLFIRINOX, mFOLFIRINOX, and NALIRIFOX all as “Preferred” category 1 regimens for metastatic disease with good performance status, with a footnote acknowledging that NALIRIFOX “does not appear to have an advantage over FOLFIRINOX”.

Recent head-to-head randomized trials have challenged the assumption that FOLFIRINOX is universally superior. The PASS-01 trial (2025) was the first prospective randomized trial directly comparing mFOLFIRINOX versus GnP with integrated molecular profiling in treatment-naïve metastatic PDAC; while PFS, the primary endpoint, was not significantly different between arms (4.0 vs. 5.3 months; HR 1.37; *p* = 0.069), OS was statistically significantly longer with GnP (9.7 vs. 8.5 months; HR 1.57; *p* = 0.017) [9]. Notably, the second-line setting appeared inadequate for precision-guided therapy: median time on second-line treatment was only 2.1 months, and correlate-guided second-line choices did not improve OS compared with standard chemotherapy (5.4 vs. 4.4 months; *p* = 0.45), suggesting that biomarker-informed selection must occur at the first-line stage rather than being deferred to later lines [9]. Notably, PASS-01 was a phase II trial powered for PFS rather than OS, and the OS difference favoring GnP was a secondary analysis without adjustment for multiple comparisons, warranting interpretation as hypothesis-generating rather than definitive.

The GENERATE trial (JCOG1611, 2025), a Japanese randomized phase II/III trial that enrolled 527 patients, was stopped early for futility of the mFOLFIRINOX arm, with GnP demonstrating superior OS (17.1 versus 14.0 months; HR 1.31; 95% confidence interval (CI) 0.97–1.77) and less gastrointestinal toxicity [10]. However, the GENERATE trial warrants cautious interpretation. It was conducted exclusively in Japan, where uridine diphosphate glucuronosyltransferase 1A1 (*UGT1A1)* polymorphisms affecting irinotecan metabolism are more prevalent, and early stopping at an interim analysis may overestimate treatment effects [10].

Indirect comparisons have yielded mixed results. A Lancet Oncology Bayesian network meta-analysis (2024) pooling 79 randomized trials (22,168 patients) [11] suggested FOLFIRINOX had a modest PFS advantage over GnP (HR 0.70, 95% CI 0.56–0.88), but this did not translate to a clear OS benefit (HR 0.83, 95% CI 0.65–1.04) [11]. Notably, the authors identified the GENERATE trial as “a key determinant for the inconsistency between the direct and indirect evidence” for the FOLFIRINOX versus GnP comparison [11]. A systematic review and meta-analysis by Nichetti et al. (2024) found no significant difference in OS between FOLFIRINOX and NALIRIFOX (HR 1.06, 95% CI 0.81–1.39; *p* = 0.65), though GnP had inferior OS compared with NALIRIFOX (HR 1.18, 95% CI 1.00–1.39; *p* = 0.05) [1,12]. The 2025 Lancet seminar by Stoop et al., incorporating reconstructed individual patient data, similarly concluded that no significant OS difference exists between FOLFIRINOX/mFOLFIRINOX and GnP [1].

A large real-world cohort study published in JAMA Network Open (2022) showed that FOLFIRINOX was associated with approximately 2.3 months longer survival. Although the authors employed propensity score matching to mitigate confounding, the subsequent randomized data from PASS-01 and GENERATE suggest that residual selection bias, with fitter patients preferentially receiving FOLFIRINOX, likely contributed to the observed advantage [13]. The reconciliation of these data is straightforward: based on available randomized data, when selection bias is eliminated through randomization, the two regimens appear equivalent, or GnP may be superior. This finding provides the strongest argument for biomarker-guided selection, that if regimens are equivalent overall, identifying molecular subgroups with differential benefit becomes clinically essential.

## 4. Molecular Landscape of Metastatic PDAC

### 4.1. Core Driver Mutations

The genomic architecture of PDAC is dominated by four recurrent driver events that accumulate during the stepwise progression from pancreatic intraepithelial neoplasia (PanIN) to invasive cancer [1,14]. Kirsten rat sarcoma viral oncogene homolog (*KRAS*) activating mutations occur as the earliest genetic event and are found in approximately 88–95% of PDACs [1,8,14]. As PanIN progresses, additional inactivating mutations in tumor suppressor genes are acquired: tumor protein p53 (*TP53*) is the most frequently co-mutated tumor suppressor, followed by cyclin-dependent kinase inhibitor 2A (*CDKN2A*), inactivated through point mutations, homozygous deletions, or promoter methylation, and SMAD family member 4 (*SMAD4*) [1,8,14,15].

The co-occurrence of multiple driver mutations has prognostic significance: tumors harboring all four driver alterations (*KRAS + TP53 + CDKN2A + SMAD4*) tend to behave more aggressively [1,14]. Additional recurrent genomic events include AT-rich interaction domain 1A (*ARID1A*) alteration, ring finger protein 43 (*RNF43*) mutation, methylthioadenosine phosphorylase (*MTAP*) deletion, transforming growth factor beta receptor 2 (*TGFBR2*) loss, and *MYC* proto-oncogene amplification [8]. While genomic features provide biologic context for biomarker development, most have not yet been validated for regimen selection on their own.

*KRAS* remains the foundational oncogenic driver in PDAC. The most common *KRAS* variants are G12D (41–44%), G12V (30–33%), and G12R (13–16%), with Q61H (4–6%) and the rare but targetable G12C (1–2%) accounting for the remainder [1,15]. Although the updated COMPASS study showed that *KRAS* allelic imbalance, defined as amplification of the mutant copy relative to the wild-type allele, is enriched in basal-like tumors, specific *KRAS* mutants and allelic states alone were not independently prognostic or predictive for regimen selection [8]. Notably, the largest real-world analysis to date (*n* = 8743) found that the prognostic impact of *KRAS* allele type was restricted to classical-subtype tumors; in strongly basal tumors, all mutant *KRAS* alleles had similarly poor outcomes [16].

*KRAS* wild-type tumors represent approximately 8–10% of cases and are enriched for alternative actionable drivers such as neurotrophic tyrosine receptor kinase (*NTRK*) fusions, v-Raf murine sarcoma viral oncogene homolog B (*BRAF*) V600E mutations, rearranged during transfection (*RET*) fusions, fibroblast growth factor receptor 2 (*FGFR2*) fusions, or neuregulin 1 (*NRG1*) fusions, which may redirect treatment toward targeted therapy rather than standard chemotherapy [15,17]. The NCCN guidelines now recommend comprehensive tumor/somatic molecular profiling, preferably including both DNA and RNA sequencing, for all patients who are candidates for anti-cancer therapy, specifically to identify these actionable alterations.

### 4.2. Transcriptomic Subtypes

Transcriptomic studies have added a second layer of clinically relevant classification. Across the systems proposed by Collisson (2011), Moffitt (2015), and Bailey (2016), the most reproducible high-level dichotomy is between classical and basal-like (also termed quasi-mesenchymal or squamous-like) disease [18,19,20].

Classical tumors retain a more epithelial and differentiated gene expression program, characterized by high expression of transcription factors such as GATA-binding protein 6 (*GATA6*). These tumors are generally associated with better prognosis and, in observational studies, appear to respond better to chemotherapy [8,18,19,20,21]. Basal-like tumors exhibit a more mesenchymal and undifferentiated program, with loss of epithelial markers and upregulation of genes associated with epithelial-to-mesenchymal transition (EMT). These tumors are associated with more aggressive behavior, poorer survival, and relative chemotherapeutic refractoriness [8,18,19,20]. In the updated COMPASS analysis, approximately one-fifth of sequenced tumors were basal-like, and this subtype remained an adverse prognostic factor even after broader molecular characterization [8].

The largest real-world molecular comparison (Caris Life Sciences, *n* = 8743) confirmed that strongly basal tumors had higher rates of *KRAS*, *TP53*, and *ARID1A* mutations, while SMAD4 mutations were more common in classical tumors. Basal tumors also showed transcriptional evidence of an inflamed but immunosuppressive microenvironment, with higher programmed death-ligand 1 (PD-L1) expression and upregulation of immune exhaustion genes (cytotoxic T-lymphocyte-associated protein 4 [*CTLA4*], T-cell immunoglobulin and mucin domain-containing protein 3 [*TIM3*], and programmed cell death protein 1 [PD-1]) [16].

From a practical standpoint, the Purity Independent Subtyping of Tumors (PurIST) classifier has emerged as the most clinically deployable transcriptomic tool, classifying tumors into classical versus basal-like subtypes [9,22]. Unlike earlier classifiers that required high tumor purity, PurIST uses focused gene expression ratios that perform reliably regardless of stromal contamination, a critical advantage for metastatic biopsies, which often have low tumor cellularity [9,22]. The predictive value of transcriptomic subtype for regimen selection is discussed below.

## 5. Biomarkers Favoring FOLFIRINOX

Because FOLFIRINOX contains oxaliplatin, the strongest treatment-specific biomarker literature in PDAC has centered on DNA repair deficiency and platinum sensitivity. The evidence is more compelling here than for any other biomarker class reviewed in this manuscript.

Within this section, it is useful to separate biomarkers that actively support early platinum exposure from those that are better understood as enrichment markers among patients already selected for FOLFIRINOX. Homologous recombination deficiency (HRD)-related biomarkers come closest to clinically actionable predictors of regimen benefit, whereas transcriptomic and *KRAS*-based signals remain supportive and hypothesis-generating rather than practice-defining [8,23,24].

### 5.1. Homologous Recombination Repair Deficiency

Germline *BRCA1*, *BRCA2*, and *PALB2* alterations remain the clearest clinically actionable predictors of platinum sensitivity in PDAC, and current NCCN guidelines now recommend germline testing for all pancreatic cancer patients regardless of family history. In a matched retrospective study of patients receiving platinum-based therapy, Wattenberg and colleagues reported an objective response rate of 58% in germline *BRCA1/2*- or *PALB2*-mutated PDAC, compared with 21% in controls, and a longer real-world progression-free survival of 10.1 versus 6.9 months [23]. These data are consistent with the biologic expectation that impaired double-strand break repair confers sensitivity to platinum exposure.

Importantly, homologous recombination repair (HRR) biology likely extends beyond *BRCA1/2* and *PALB2* alone. Additional genes implicated in HRR and Fanconi anemia pathways, including *ATM*, checkpoint kinase 2(*CHEK2*), *RAD51*-family genes, and Fanconi anemia complementation group A (*FANCA*), may broaden the population with platinum-sensitive biology, although the predictive significance of each gene remains less certain than for *BRCA1/2* or *PALB2* [25]. Among the *RAD51* paralogs, *RAD51C* participates in protein complexes essential for loading RAD51 onto damaged DNA during homologous recombination. An exceptional response to FOLFIRINOX has been reported in a patient with metastatic PDAC harboring a germline *RAD51C* mutation, although this observation is limited to a single case report [26]. One retrospective analysis found that DDR gene mutations overall (beyond *BRCA/PALB2*) were associated with improved PFS (9.95 vs. 6.51 months) and OS (20.5 vs. 16.8 months) on platinum therapy [27]. In clinical practice, these broader alterations are best interpreted in the context of the total genomic profile rather than as isolated binary markers. Reflecting growing confidence in this biomarker-treatment pairing, NCCN now includes cisplatin/gemcitabine as a preferred option for patients with known *BRCA1/2* or *PALB2* mutations.

From a practical standpoint, the value of HRR testing is not simply prognostic; it also informs therapeutic sequencing. In fit patients, identification of a *BRCA1/2* or *PALB2* alteration argues strongly for early platinum exposure to maximize initial disease control and to preserve the option of poly(ADP-ribose) polymerase (PARP)inhibitor maintenance in selected patients [23,28]. The POLO trial demonstrated a significant PFS benefit with maintenance olaparib versus placebo after platinum-based therapy in germline *BRCA*-mutated PDAC (median PFS 7.4 vs. 3.8 months; HR 0.53; *p* = 0.004), although the final OS analysis showed no statistically significant difference [28]. The phase 2 POLAR trial (NCT04666740) extended this paradigm by evaluating maintenance pembrolizumab plus olaparib following platinum-based chemotherapy in HRD-stratified metastatic PDAC. In the *BRCA1/2*- or *PALB2*-mutated cohort (*n* = 33), median OS was 28 months with a 3-year OS rate of 44%. Circulating tumor DNA(ctDNA) response and frameshift indel neoantigen enrichment were associated with durable benefit, reinforcing that HRD testing should inform maintenance strategy beyond platinum choice alone [29]. HRD testing should therefore inform both first-line chemotherapy selection and maintenance strategy. PARP ± immune checkpoint blockade combinations now represent biologically rational options for patients with core HRD alterations [28,29].

The Center for Cancer Genomics and Advanced Therapeutics (C-CAT) database study (4356 patients) adds important nuance. In patients with *BRCA2* pathogenic variants (3.3%), FOLFIRINOX demonstrated a higher response rate (66.7% vs. 33.3%) and longer time to treatment failure compared with GnP. However, OS was comparable between regimens (HR 1.11, *p* = 0.665) [30]. This finding is explained by treatment sequencing: patients who started with GnP and received FOLFIRINOX as second-line therapy achieved similar survival to those receiving FOLFIRINOX upfront. This underscores the importance of ensuring platinum exposure at some point during the treatment course rather than necessarily as first-line therapy. Germline BRCA1/2 and PALB2 alterations currently represent the strongest predictive biomarkers for platinum sensitivity in PDAC and also serve a therapeutic role by identifying candidates for PARP inhibitor maintenance.

### 5.2. Genomic Scar Signatures

While Section 5.1 addressed HRD identified through specific gene mutations, a substantial proportion of HRD tumors arise from mechanisms not captured by standard gene panel testing, including epigenetic silencing (e.g., *BRCA1* promoter methylation), biallelic loss through structural events, and unknown mechanisms [8,24]. Genomic scar signatures were developed to detect the cumulative consequences of defective DNA repair, regardless of the underlying cause [24,31].

Scar-based measures capture the genomic consequences of defective DNA repair, including loss of heterozygosity (LOH), telomeric allelic imbalance (TAI), and large-scale state transitions (LST), and may identify platinum-sensitive tumors missed by conventional mutation testing. Chen et al. found that HRD signature (HRDsig)positive tumors (9% of metastatic PDAC) had dramatically better outcomes on FOLFIRINOX versus GnP (HR 0.37, 95% CI 0.22–0.62), with median OS of 12.8 versus 4.5 months [24]. The HRDsig is a machine-learning-based algorithm that incorporates copy-number and indel features, rather than relying solely on HRR gene alterations, enabling detection of HRD arising from genomic or epigenetic mechanisms beyond BRCA mutations [31].

The updated COMPASS dataset confirmed that HRDetect-high tumors form a distinct subgroup with improved mFOLFIRINOX response [8]. A Memorial Sloan Kettering retrospective analysis using genomic HRD assessment (core homologous recombination mutations and/or biallelic loss) found that HRD-positive patients had significantly improved PFS on first-line platinum-based therapy (HR 0.44, *p* < 0.01), supporting the clinical utility of genomic scar-based approaches beyond single-gene testing [32].

As discussed in Section 5.1, the therapeutic sequencing implications of HRD identification extend beyond first-line regimen selection, with PARP-based maintenance strategies now supported by the POLO and POLAR trials [28,29]. Scar-based signatures may broaden the population eligible for this treatment paradigm by identifying HRD tumors not captured by germline testing alone, although this application has not yet been prospectively validated.

These observations are important because many clinically relevant HRD tumors are not captured by germline testing alone. Scar-based metrics integrate the cumulative genomic consequences of defective DNA repair and may therefore identify a broader platinum-sensitive phenotype than single-gene assays [8,24]. At the same time, this remains an evolving area. Different platforms use different algorithms, thresholds, and tissue requirements, and concordance among HRDsig, HRDetect, and panel-based approaches is not yet standardized [8,24]. Furthermore, all scar-based HRD studies in PDAC to date are retrospective, and no prospective trial has yet used a genomic scar signature as a stratification biomarker for regimen selection. For now, scar signatures are best viewed as tools that can strengthen a platinum-favoring decision rather than as standalone replacements for careful genomic interpretation. Genomic scar signatures, therefore, function as exploratory predictive biomarkers that may identify a broader platinum-sensitive population beyond single-gene testing, though all available data remain retrospective, and no prospective trial has yet used a genomic scar signature as a stratification biomarker for regimen selection.

### 5.3. Transcriptomic Subtypes and GATA6

As noted above, the classical versus basal-like transcriptomic dichotomy has emerged as a clinically relevant classification in PDAC. Observational data consistently show that classical tumors have better outcomes on FOLFIRINOX. Lansbergen et al. found that classical tumors (by PurIST) had longer PFS (216 vs. 78 days) and OS on FOLFIRINOX than basal-like tumors [21]. Wenric et al. (JCO Precision Oncology, 2025) showed that classical patients with ECOG PS 0–1 had a 33% relative risk reduction in death with FOLFIRINOX versus GnP (HR 0.67, *p* = 0.009) [22].

*GATA6* immunohistochemistry (IHC) serves as a practical surrogate for transcriptomic subtype: *GATA6*-low tumors had markedly shorter OS on FOLFIRINOX (323 vs. 746 days), while untreated patients showed no difference, suggesting a predictive rather than purely prognostic role [21]. A 2024 multi-center analysis combining the Canadian COMPASS cohort and the Italian IRCCS Fondazione Policlinico cohort (*n* = 465) confirmed *GATA6* as both prognostic and predictive of mFOLFIRINOX response, with GATA6-low tumors showing significantly less benefit from mFOLFIRINOX (interaction *p* = 0.003), leading the authors to suggest that *GATA6*-low tumors should receive first-line GnP-based regimens [33].

However, *GATA6* knockdown in organoid models did not consistently recapitulate chemotherapy resistance, implying that *GATA6* functions as a surrogate of broader biology rather than a causal driver of response [21].

Critically, the PASS-01 trial found the opposite of observational data: classical tumors appeared to do better on GnP than mFOLFIRINOX (OS 13.9 vs. 9.7 months, *p* = 0.047 [9]. This paradox likely reflects selection bias in observational studies. Notably, PASS-01 excluded germline BRCA1/2 and PALB2 carriers, thereby depleting the HRD subgroup most likely to benefit from platinum/FOLFIRINOX, which may partly explain the unexpected result [9].

Whether *GATA6* status predicts differential benefit between FOLFIRINOX and GnP, as opposed to general chemotherapy responsiveness, remains to be tested in further randomized comparisons.

Biologically, this signal is plausible. Classical tumors tend to preserve epithelial differentiation and secretory programs, whereas basal-like tumors show dedifferentiation, EMT features, and broader treatment resistance [8,18,20,21]. However, current evidence does not justify using transcriptomic subtypes in isolation to select or deny FOLFIRINOX to an otherwise fit patient. Rather, subtype and *GATA6* are best interpreted as contextual markers: they may strengthen confidence in a FOLFIRINOX-based choice when they align with HRD or other platinum-favoring features, but they are not yet sufficiently settled to override randomized trial uncertainty [9,21,22].In summary, *GATA6* expression shows evidence of both prognostic and predictive value in retrospective data, but conflicting results from the PASS-01 randomized trial mean that transcriptomic subtype currently remains an exploratory rather than validated predictive biomarker for regimen selection.

### 5.4. KRAS Mutation Subtypes

For the majority of *KRAS*-mutated PDAC patients (G12D, G12V, G12R, collectively comprising approximately 85–90% of *KRAS*-mutant tumors), the available retrospective data suggest that FOLFIRINOX may be the preferred regimen when patient fitness permits [34]. A large clinicogenomic analysis (*n* = 2433 metastatic PDAC patients) published in JAMA Network Open found that *KRAS* G12D and G12V were associated with significantly worse outcomes compared to *KRAS* wild-type, while *KRAS* G12R was associated with more favorable outcomes. FOLFIRINOX was associated with numerically improved time to next treatment (TTNT) and OS compared with GnP across most *KRAS* subtypes [34].

For the rare *KRAS* G12C subgroup (~2%), the optimal first-line regimen remains uncertain. One large analysis showed significantly longer OS with GnP over FOLFIRINOX (470 vs. 240 days, HR 0.32, *p* = 0.013) [35], while the Norton et al. analysis found FOLFIRINOX associated with the longest TTNT across all G12 variants, including G12C [34]. These conflicting results likely reflect small sample sizes within the G12C subgroup and differences in patient selection across datasets. Prospective validation in *KRAS* subtype-stratified trials is needed before recommending variant-specific regimen selection. Importantly, *KRAS* G12C status also identifies patients eligible for targeted therapy with adagrasib or sotorasib upon disease progression, adding further value to detailed *KRAS* characterization [15,17].

At present, *KRAS* subtyping should be interpreted as an emerging refinement rather than a primary regimen selector. The retrospective signal favoring FOLFIRINOX across common *KRAS* variants is intriguing because it suggests that not all *KRAS*-mutated PDAC behaves identically, yet the available data remain vulnerable to confounding by performance status and treatment selection [34,35]. The immediate clinical value of detailed *KRAS* characterization is twofold: first, to recognize that common non-G12C variants do not currently redirect therapy away from chemotherapy; and second, to identify the rare *KRAS* G12C subgroup or *KRAS*-wild-type tumors in whom targeted options or alternative oncogenic drivers may become clinically relevant [15,34,35]. However, the therapeutic landscape for common *KRAS* variants is rapidly evolving. Daraxonrasib (RMC-6236), a first-in-class pan-*RAS*(ON) multi-selective tri-complex inhibitor targeting active guanosine triphosphate (GTP)-bound *KRAS* (including G12D, G12V, and G12R), demonstrated an ORR of 20% and a disease control rate of 87% in heavily pretreated metastatic PDAC in phase 1 [36]. A randomized phase 3 trial is now evaluating daraxonrasib alone or in combination with GnP versus standard chemotherapy in first-line metastatic PDAC (NCT07491445). If positive, this trial could fundamentally redefine *KRAS* mutation status from a prognostic marker to a primary treatment-selection biomarker, directing patients toward targeted therapy rather than empirical chemotherapy [36,37].

## 6. Biomarkers Favoring Gemcitabine Plus Nab-Paclitaxel

Compared with the FOLFIRINOX literature, biomarker data for GnP are less mature and more heterogeneous. Nevertheless, several candidate markers have emerged that directly address the question of first-line regimen choice.

### 6.1. Human Equilibrative Nucleoside Transporter 1 (hENT1)

hENT1 is biologically attractive as a biomarker for the gemcitabine component of GnP because gemcitabine is a nucleoside analog that requires active transport into cells to exert its cytotoxic effect [38,39]. Tumors with low hENT1 expression cannot efficiently take up gemcitabine, rendering the drug less effective regardless of dose intensity [39].

The strongest evidence for hENT1 as a predictive biomarker comes from the COMPASS biomarker study published by Perera and colleagues in Clinical Cancer Research in 2022 [40]. In this prospective observational analysis of 253 patients, the investigators demonstrated that hENT1 expression is a treatment-specific predictive biomarker for GnP but not for mFOLFIRINOX. Among patients treated with GnP, hENT1-high tumors had significantly higher response rates (43% vs. 21%, *p* = 0.038) and longer median overall survival (10.6 vs. 6.7 months, *p* = 0.001). In contrast, among patients treated with mFOLFIRINOX, there was no difference in response rate (35% vs. 28%, *p* = 0.56) or overall survival (10.6 vs. 10.5 months, *p* = 0.45). Critically, the treatment-by-biomarker interaction was statistically significant (*p* = 0.002), confirming that hENT1 is a true predictive marker rather than simply a prognostic factor [40]. However, prospective validation in a randomized biomarker-stratified trial is still lacking.

Sahin and colleagues independently validated these findings in a retrospective study of 106 patients with advanced PDAC [41]. The investigators examined both hENT1 and *TUBB3* expression and found that a combined biomarker score (*TUBB3*-low/hENT1-high) was strongly predictive of GnP benefit, with an odds ratio for disease control of 11.96 (95% CI 2.61–54.82, *p* = 0.001) and a HR for PFS of 0.33 (95% CI 0.18–0.60, *p* =0.001). Importantly, no differences in response rates or PFS were observed among patients receiving FOLFIRINOX, reinforcing the treatment-specific nature of these biomarkers [41]. The combination of hENT1 and *TUBB3* may therefore provide a practical immunohistochemistry-based approach for identifying patients most likely to benefit from GnP.

### 6.2. Class III β-Tubulin (TUBB3)

*TUBB3* is a microtubule component implicated in taxane resistance across multiple tumor types [42]. Nab-paclitaxel works by stabilizing microtubules and preventing cell division. Mechanistic studies have shown that *TUBB3* overexpression alters microtubule dynamics and reduces paclitaxel’s ability to stabilize microtubules, thereby conferring resistance [43]. Conversely, tumors lacking *TUBB3* are more susceptible to taxane-induced cell death, making *TUBB3* absence a logical biomarker for GnP sensitivity. The initial evidence for *TUBB3* as a GnP biomarker came from a retrospective Japanese study by Kato and colleagues published in Human Pathology in 2018 [44]. The investigators reviewed 75 patients with unresectable PDAC who received GnP, with 67 analyzable specimens available for *TUBB3* IHC staining. Of these, 14 (21%) were negative for *TUBB3*, and 53 (79%) were positive. The absence of *TUBB3* expression was associated with significantly better outcomes: the disease control rate was 100% versus 64.2% (*p* = 0.008), and the median PFS was 7.1 versus 3.7 months (*p* = 0.036). In multivariate analysis, negative *TUBB3* expression remained an independent predictor of prolonged PFS (HR 2.41, 95% CI 1.11–5.24, *p* = 0.026) [44].

The same group published a follow-up study in Pancreas in 2022 that expanded the analysis to 113 patients receiving either GnP or FOLFIRINOX as first-line therapy [45]. This study confirmed that high *TUBB3* expression was associated with lower disease control rate (*p* = 0.017) and shorter PFS (*p* = 0.019) in the GnP group, and that *TUBB3* was an independent predictor of PFS on multivariate analysis (*p* = 0.045). Critically, *TUBB3* expression was not correlated with PFS or OS in the FOLFIRINOX group, confirming its treatment-specific predictive value for GnP rather than general prognostic significance [45].

However, several limitations should be acknowledged. Both studies were single-center, retrospective, and conducted in Japanese populations, and the combined sample size remains modest (*n* = 113 in the larger study). *TUBB3* IHC scoring thresholds have not been standardized across laboratories, and no prospective validation study has been completed. Additionally, the studies enrolled mixed populations of patients with metastatic and locally advanced disease, which may limit their direct applicability to purely metastatic decision-making.

### 6.3. Secreted Protein Acidic and Rich in Cysteine (SPARC): A Negative Biomarker

*SPARC* was initially hypothesized to enhance nab-paclitaxel delivery through albumin-*SPARC* binding interactions in the tumor stroma, raising interest in *SPARC* as a predictive biomarker for GnP benefit [46,47,48].

This hypothesis was tested in an exploratory analysis of the phase III MPACT trial, published by Hidalgo and colleagues in Clinical Cancer Research in 2015 [49]. Among 256 patients with evaluable stromal *SPARC* expression, no association was found between *SPARC* and clinical outcomes. There was no difference in OS by *SPARC* status (*p* = 0.73), no difference in PFS (*p* = 0.85), and no association between stromal *SPARC* and ORR (*p* = 0.61). Furthermore, there was no treatment-by-biomarker interaction in either the nab-paclitaxel/gemcitabine arm or the gemcitabine monotherapy arm. The authors concluded that stromal *SPARC* expression should not be used to select patients for nab-paclitaxel-based therapy [49].

The *SPARC* story illustrates that biologic plausibility alone is insufficient for biomarker validation. Despite a compelling mechanistic hypothesis, the clinical data did not support *SPARC* as a predictive marker.

### 6.4. MCL-1 Expression

In a retrospective study of 38 patients with metastatic PDAC treated with first-line GnP, Urabe and colleagues found that myeloid cell leukemia 1 (*MCL-1*) positive tumors (>20% immunoreactivity) had a significantly higher disease control rate (95.7% vs. 73.3%, *p* = 0.046), longer PFS (7.2 vs. 4.9 months, *p* = 0.018), and longer OS (14.9 vs. 9.2 months, *p* = 0.008) compared with *MCL-1*-negative tumors, with *MCL-1* expression remaining an independent predictor on multivariate analysis [50]. *MCL-1* is an anti-apoptotic B-cell lymphoma 2 (*BCL-2*) family protein; its paradoxical association with better GnP response may reflect that nab-paclitaxel-induced mitotic arrest preferentially triggers *MCL*-*1*-dependent apoptotic pathways in *MCL-1*-expressing cells, although this mechanism remains speculative [50]. The study is limited by its small sample size (*n* = 38) and single-center retrospective design, and it did not include a FOLFIRINOX comparator arm. Without a comparator arm, it is impossible to determine whether *MCL-1* is truly predictive for GnP specifically or merely prognostic overall. MCL-1 should therefore be classified as indeterminate (prognostic versus predictive unclear) pending studies that include a FOLFIRINOX comparator.

### 6.5. Pathway-Specific Mutations: NOTCH, KIT, and PI3K

Beyond individual gene alterations, pathway-level analysis may provide additional predictive information for regimen selection. The NOTCH signaling pathway plays an important role in pancreatic cancer development. *NOTCH* receptors (*NOTCH1–4*) and their ligands regulate cell proliferation, differentiation, and apoptosis, and dysregulation of this pathway has been implicated in PDAC tumorigenesis and progression [51].

In a retrospective study of 142 patients with unresectable stage III/IV PDAC, Paredes de la Fuente and colleagues examined the association between molecular pathway mutations and outcomes on first-line FOLFIRINOX or GnP [52]. At the overall cohort level, *NOTCH*-pathway mutations were associated with longer median OS compared with pathway-wild-type tumors (15.0 vs. 12.3 months, *p* = 0.007), and *KIT*-pathway mutations with even longer median OS (21.3 vs. 12.1 months, *p* = 0.04).

Most importantly for regimen selection, the treatment-specific subgroup analysis suggested a GnP-favoring signal. Within the GnP cohort, *PI3K*-pathway mutations were associated with longer PFS (6.6 vs. 5.7 months, *p* = 0.03), and *KIT*-pathway mutations with longer PFS (10.3 vs. 6.2 months, *p* = 0.03) [52]. These associations were not observed in the FOLFIRINOX subgroup, suggesting a treatment-specific rather than purely prognostic effect.

This study is exploratory and limited by its single-center retrospective design, the inclusion of both locally advanced and metastatic disease, and the broad grouping of heterogeneous genomic events into pathway-level categories. Additionally, the absolute PFS differences for *PI3K*-pathway mutations were modes, and the study was not powered for formal treatment-by-biomarker interaction testing. Nevertheless, it provides one of the few currently available examples of a pathway-specific signal that may preferentially support GnP over FOLFIRINOX, warranting prospective validation. These pathway-level signals are best classified as exploratory predictive biomarkers that require confirmation in larger, independent cohorts before clinical application.

**Figure 1:**
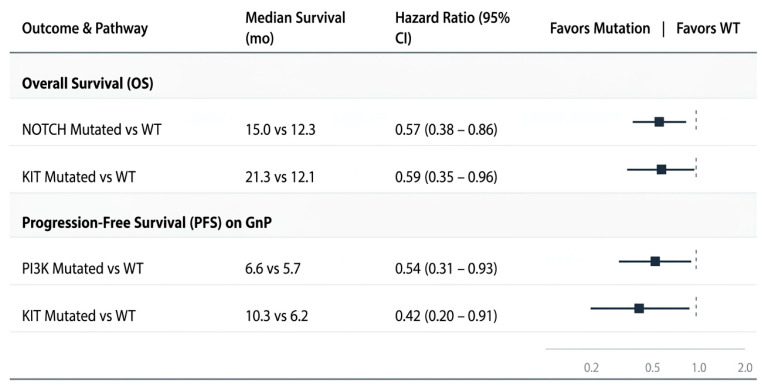
Survival outcomes associated with pathway-specific mutations in advanced PDAC. *NOTCH* and *KIT* pathway mutations were associated with longer OS in the full cohort, and *PI3K* and *KIT* pathway mutations were associated with longer PFS in the GnP subgroup. Data summarized from Paredes de la Fuente et al. [52].

These pathway-specific associations are summarized in Figure 1.

### 6.6. SMAD4 Alterations

*SMAD4* is a central mediator of the transforming growth factor beta (*TGF-β*) signaling pathway and is inactivated in approximately 20–55% of PDACs through homozygous deletion, intragenic mutation, or loss of heterozygosity [1]. Beyond its established role as a tumor suppressor and prognostic marker associated with metastatic propensity, emerging evidence suggests that *SMAD4* status may function as a treatment-specific predictive biomarker for regimen selection. In a study of 322 patients with localized PDAC receiving neoadjuvant chemotherapy, Ecker et al. found that SMAD4 alterations were associated with metastatic progression on FOLFIRINOX (30.0% vs. 14.5%; *p* = 0.009) and lower resection rates (37.1% vs. 66.7%; *p* < 0.001). No such associations were observed in GnP-treated patients (*p* = 0.866 and *p* = 0.605, respectively) [53]. A multicenter retrospective cohort (*n* = 311) confirmed that *SMAD4* alterations predicted metastatic progression (odds ratio 1.89; *p* = 0.047) and failure to complete surgical resection (odds ratio 0.49; *p* = 0.024) only among FOLFIRINOX-treated patients, with no association in the GnP group [54]. Bioinformatic analyses of PDAC cell lines and genomic datasets have further suggested that *SMAD4*-deleted tumors may retain sensitivity to gemcitabine through upregulation of cell cycle-related pathways, providing a mechanistic rationale for the treatment-specific clinical observations [55]. These data collectively suggest that *SMAD4* loss may function as a biomarker of FOLFIRINOX resistance rather than general chemoresistance, potentially favoring GnP-based therapy in *SMAD4*-altered tumors. However, all available clinical data are retrospective and derived from the localized/neoadjuvant setting rather than metastatic disease, and prospective validation in the metastatic population is needed before *SMAD4* status can be used for first-line regimen selection. *SMAD4* alteration is therefore classified as an exploratory predictive biomarker, with the additional caveat that all clinical data derive from the localized/neoadjuvant setting rather than metastatic disease.

A comprehensive summary of all candidate biomarkers for regimen selection, including their key findings, evidence level, and regimen signal, is presented in Table 1.

## 7. Emerging Integrative Profiling Approaches and Future Directions

The updated COMPASS analysis demonstrated that HRD signatures, transcriptomic subtype, *KRAS* dosage, inflammatory status, and tumor microenvironmental features can coexist within the same patient and jointly shape outcome [8]. This complexity explains why individual mutations often perform poorly as universal treatment selectors and supports the development of integrated, multi-marker models for regimen selection.

A major practical barrier is tissue quality. In the Lansbergen metastatic cohort, roughly half of the biopsies were unsuitable for high-quality RNA-based classification, highlighting the need for formalin-fixed paraffin-embedded (FFPE)-compatible surrogates such as *GATA6* IHC [21].

### 7.1. AI-Enhanced Transcriptomic Tools

Artificial intelligence (AI)-enhanced transcriptomic tools are emerging as potential solutions to this challenge, specifically because they generate drug-specific rather than purely prognostic predictions. The GemPred classifier was developed in the adjuvant setting to predict gemcitabine sensitivity. GemPred-positive patients receiving adjuvant gemcitabine had dramatically improved outcomes compared with GemPred-negative patients (91.3 vs. 33 months OS; HR 0.403, 95% CI 0.221–0.735, *p* = 0.002), suggesting it could identify patients most likely to benefit from gemcitabine-based therapy; however, its applicability to metastatic GnP is extrapolated and has not yet been validated in the metastatic setting [56]. GemCore, a related gemcitabine sensitivity signature, has been validated in both resected and metastatic tumors and is compatible with small biopsies, potentially bridging the gap between adjuvant-derived tools and metastatic clinical application; however, prospective validation for regimen selection is still needed [56]. The Pancreas-View platform integrates drug-specific transcriptomic signatures, including predictions for 5-fluorouracil, gemcitabine, and platinum sensitivity into a single report, enabling simultaneous assessment of likely benefit from both FOLFIRINOX and GnP components [57]. Like GemPred, Pancreas-View was developed and validated in the adjuvant setting, and prospective validation in metastatic disease is needed before these tools can inform first-line regimen selection. If validated prospectively, these tools could enable direct, biology-informed regimen selection rather than relying on single-marker surrogates. However, these tools remain investigational and face practical challenges, including the need for standardized RNA extraction workflows, validation across diverse FFPE specimen types, and reproducibility across independent laboratories.

### 7.2. Circulating Tumor DNA

ctDNA may complement tissue-based biomarkers by enabling non-invasive molecular profiling and dynamic treatment adaptation. While ctDNA is not currently validated for first-line regimen selection between FOLFIRINOX and GnP, early ctDNA clearance during therapy has been associated with improved outcomes [58]. More recently, Steiniche et al. evaluated ctDNA response evaluation criteria in solid tumors (ctDNA-RECIST) in 220 patients with metastatic PDAC and found that ctDNA maximal response before the second treatment cycle was associated with a median OS of 11.9 months versus 3.6 months for ctDNA progressive disease [59]. These data strengthen the case for ctDNA as a dynamic monitoring tool that could eventually guide early regimen switching in patients who are not responding to their initial regimen. Despite this promise, ctDNA-based approaches in PDAC face challenges, including a lack of standardized assay platforms and response thresholds, and the absence of prospective trials demonstrating that ctDNA-guided treatment switching improves survival outcomes.

### 7.3. Patient-Derived Organoid (PDO) Pharmacotyping

While the PASS-01 trial has reported its primary clinical and transcriptomic results, which showed that correlate-guided second-line therapy did not improve OS compared with standard chemotherapy, the PDO pharmacotyping correlative analyses, designed to evaluate whether ex vivo drug sensitivity testing can predict first-line regimen benefit, have not yet been published [9].

Beyond PASS-01, early-phase studies have demonstrated that PDO pharmacotyping correlates with clinical treatment response in pancreatic cancer, with reported sensitivity of up to 83% and specificity of 93% in predicting chemotherapy outcomes, supporting the feasibility of functional precision oncology approaches [60]. However, no completed randomized trial has yet demonstrated that organoid-guided first-line regimen selection improves survival in metastatic PDAC. Additional studies are exploring PurIST-guided treatment allocation in first-line settings [22]. These efforts collectively represent a shift toward prospective validation of the biomarker-regimen associations described in this review. From a feasibility standpoint, organoid pharmacotyping is limited by variable establishment success rates, the time required for organoid culture (typically two to four weeks), high cost, and the need for specialized laboratory infrastructure, all of which currently restrict its applicability to selected academic centers.

### 7.4. Novel Agents with Embedded Biomarker Programs

Beyond profiling tools that guide selection among existing regimens, novel agents with embedded biomarker programs are emerging. Elraglusib (9-ING-41), a first-in-class selective glycogen synthase kinase 3 beta (*GSK-3β*) inhibitor, was evaluated in a randomized phase 2 trial (NCT03678883; *n* = 233) in which elraglusib combined with GnP improved median OS over GnP alone in previously untreated metastatic PDAC (10.1 vs. 7.2 months; HR 0.62; 95% CI 0.46–0.84; *p* = 0.01), with a 1-year survival rate of 44.1% versus 22.3%. Correlative analyses from the same trial identified treatment-specific biomarker signals, including baseline plasma C-X-C motif chemokine ligand 2 (*CXCL2*) and tumor necrosis factor-related apoptosis-inducing ligand (*TRAIL*) ligands associated with improved survival, and therapy-induced grade 3–4 neutropenia, which correlated with improved OS in the elraglusib arm (14.4 vs. 5.9 months) but not in GnP alone. Machine learning models using pre-dose plasma cytokines achieved 88% accuracy in predicting 12-month survival, specifically in the elraglusib arm, confirming treatment-specific predictive significance [61].

### 7.5. Ongoing and Planned Biomarker-Stratified Trials

Several ongoing and planned clinical trials are poised to address the key evidence gaps identified in this review. In the HRD space, the randomized phase 2 Southwest Oncology Group (SWOG) S2001 trial (NCT04548752) is comparing olaparib plus pembrolizumab versus olaparib alone as maintenance therapy in germline BRCA1/2-mutated metastatic PDAC, building on the POLO and POLAR results discussed in Section 5.1. For KRAS-targeted therapy, the phase 3 daraxonrasib trial (NCT07491445) is evaluating a pan-RAS(ON) inhibitor alone or combined with GnP versus standard chemotherapy in first-line metastatic PDAC; if positive, KRAS mutation status could become a primary treatment-selection biomarker for approximately 90% of patients [36,37]. The PANThEON trial (NCT06529809) is a randomized phase 2 study testing whether early sequential switch from mFOLFIRINOX to GnP improves outcomes, with embedded molecular, ctDNA, and radiomic biomarker analyses [62]. PurIST-guided treatment allocation studies are exploring whether transcriptomic subtype can prospectively direct first-line regimen selection [22]. The PASS-01 PDO pharmacotyping correlatives remain pending and will determine whether organoid-based drug sensitivity testing can predict regimen benefit [9]. A phase 3 elraglusib trial is being planned based on the phase 2 OS benefit with GnP [61]. Collectively, these trials represent a shift from retrospective biomarker-outcome associations toward prospective, biomarker-stratified treatment selection.

## 8. Practical Biomarker-Guided Treatment Framework

A pragmatic framework for first-line treatment selection in metastatic PDAC can be proposed based on the convergence of available evidence, even before definitive prospective validation. First, all patients should undergo comprehensive germline and somatic profiling early in the metastatic course, ideally before initiating first-line therapy. The strongest current signal favoring a platinum-containing approach is HRD, particularly germline *BRCA1/2* or *PALB2* mutations and broader HRD signatures; when the patient is fit enough, these data support use of FOLFIRINOX or another platinum-based strategy [23,28]. Importantly, HRD identification also informs maintenance strategy beyond first-line regimen selection. Olaparib monotherapy is Food and Drug Administration (FDA) approved and NCCN-preferred for maintenance in germline *BRCA1/2*-mutated metastatic PDAC following platinum-based chemotherapy, based on the phase III POLO trial [28]. The phase 2 POLAR trial (NCT04666740), a single-arm study at Memorial Sloan Kettering, has provided preliminary evidence that maintenance pembrolizumab plus olaparib may yield durable benefit in the *BRCA1/2*- or *PALB2*-mutated cohort (median OS 28 months; 3-year OS rate 44%), although the trial did not meet its co-primary endpoints and lacked a comparator arm [29]. The randomized phase 2 SWOG S2001 trial (NCT04548752), which directly compares olaparib plus pembrolizumab versus olaparib alone in germline *BRCA1/2*-mutated metastatic PDAC, is expected to provide the comparative evidence needed to determine whether adding immunotherapy to PARP inhibitor maintenance improves outcomes. These data collectively reinforce the therapeutic implications of early HRD testing that extend well beyond the initial chemotherapy choice.

Second, if HRD is absent and transcriptomic information is available, classical or *GATA6*-high disease may further support FOLFIRINOX, whereas basal-like or *GATA6*-low disease should raise concern for poorer FOLFIRINOX outcomes and may justify greater consideration of GnP. However, the conflicting PASS-01 data, in which classical tumors had better outcomes on GnP, potentially influenced by the exclusion of germline *BRCA1/2* and *PALB2* carriers and the phase II sample size, mandate caution in applying this signal outside of clinical trials [9,21]. Third, if a tumor is hENT1-high and/or *TUBB3*-low, GnP may be a biologically appealing option, though these markers are not yet mature enough to override clinical judgment [40,41]. Exploratory pathway signals such as *PI3K* or *KIT* mutations provide additional hypothesis-generating support for GnP but require prospective validation [52].

Fourth, comprehensive molecular profiling should be performed to identify the minority of patients harboring actionable alterations. These include microsatellite instability-high (MSI-H)/deficient mismatch repair (dMMR), *NTRK* fusions, *BRAF* V600E, *NRG1* fusions, rearranged during transfection (*RET*) fusions, *KRAS* G12C, and human epidermal growth factor receptor 2 *(HER2*) amplification, for whom targeted therapy or immunotherapy may entirely redirect the treatment strategy. Most of these options are currently classified by the NCCN (v1.2026) as “useful in certain circumstances “rather than preferred first-line regimens [17,63].

Importantly, none of the biomarker-regimen associations described in this framework have been tested in the context of NALIRIFOX, which is now listed as an NCCN-preferred first-line regimen. Whether these signals extend to NALIRIFOX remains an important evidence gap.

This framework may require substantial revision as novel targeted agents enter first-line evaluation. Daraxonrasib (RMC-6236), a pan-*RAS* (ON) multi-selective inhibitor, is currently being evaluated in a randomized phase 3 trial in combination with GnP versus standard chemotherapy in first-line metastatic PDAC (NCT07491445) [36,37]. If positive, *KRAS* mutation status could become a primary treatment-selection biomarker in PDAC, potentially outweighing many current chemotherapy-specific biomarker signals. Similarly, elraglusib, a *GSK-3β* inhibitor that improved OS when combined with GnP in a randomized phase 2 trial (10.1 vs. 7.2 months; HR 0.62; *p* = 0.01), is associated with treatment-specific cytokine biomarkers (*CXCL2*, *TRAIL*) that could add another predictive layer if confirmed in phase 3 [61]. These developments underscore the importance of comprehensive molecular profiling at diagnosis, as the clinical utility of the data obtained is likely to expand as new therapeutic options mature.

In the absence of a validated predictive biomarker, regimen choice should still be individualized using performance status, comorbidities, pre-existing neuropathy, cachexia and nutritional status, liver function, metastatic burden, and patient priorities. Biomarker-guided treatment in PDAC should be viewed not as an alternative to clinical judgment but as a means of making that judgment more biologically informed.

**Figure 2:**
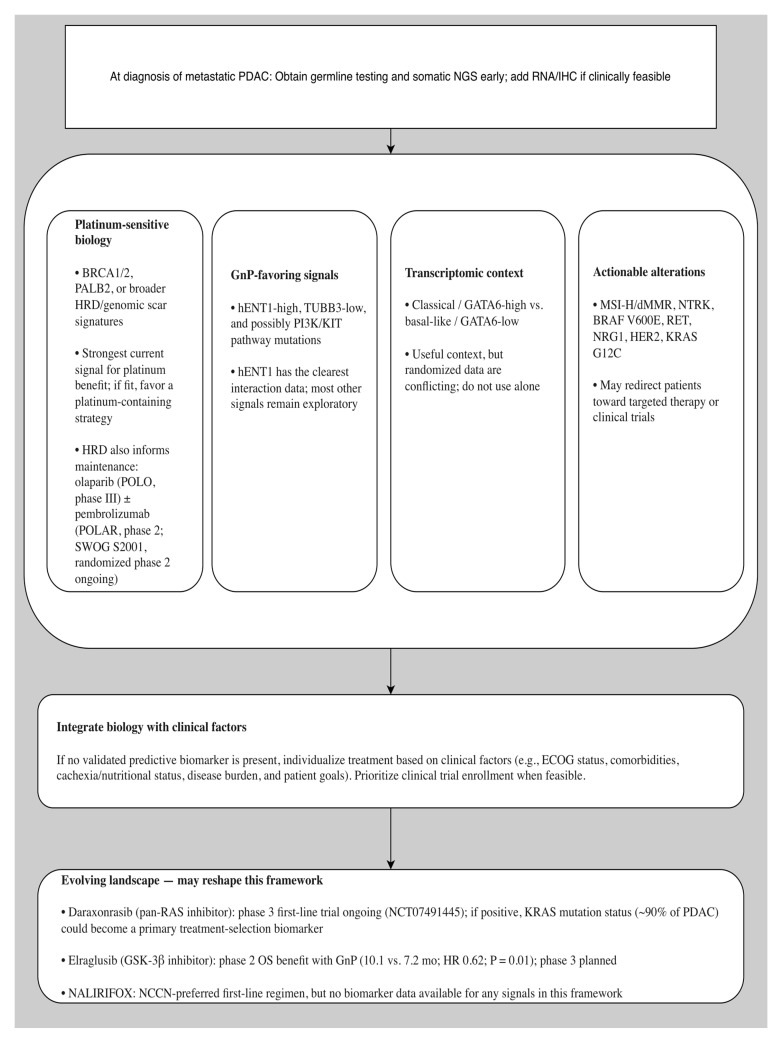
Conceptual biomarker-guided framework for first-line treatment selection in metastatic pancreatic ductal adenocarcinoma. The strongest current signal favoring a platinum-containing strategy is homologous recombination deficiency, which also informs maintenance strategy selection. hENT1, *TUBB3*, and selected pathway-level signals may support gemcitabine plus nab-paclitaxel. Transcriptomic context remains clinically relevant but not yet definitive. Actionable alterations may redirect treatment toward targeted therapy or clinical trials. Emerging agents, including pan-*RAS* inhibitors and *GSK-3β* inhibitors, may substantially revise this framework if phase 3 trials are positive. Abbreviation: NGS—next-generation sequencing.

A conceptual biomarker-guided framework for first-line regimen selection is shown in Figure 2.

## 9. Limitations and Challenges in Clinical Implementation

Several limitations currently prevent routine biomarker-based selection between FOLFIRINOX and GnP. Most of the evidence remains retrospective or observational and is therefore susceptible to treatment-selection bias. The discrepancy between observational data favoring FOLFIRINOX for classical tumors and the PASS-01 randomized finding that classical tumors had better outcomes on GnP exemplifies this challenge and underscores the risk of drawing predictive conclusions from non-randomized comparisons [9,21,22].

Biomarker assessment methodologies vary widely across studies, including germline testing, targeted DNA panels, whole-genome sequencing, RNA sequencing, and immunohistochemistry, making cross-study comparisons difficult. Many studies enrolled mixed populations of metastatic and locally advanced disease [44,45,52], which complicates interpretation for purely metastatic decision-making.

Among individual biomarkers, HRD signatures are promising but not yet standardized across platforms, with different studies employing HRDsig, HRDetect, or other algorithmic approaches [8,24]. Transcriptomic classifiers require better harmonization and FFPE-compatible workflows before routine clinical deployment. hENT1 has the strongest treatment-specific interaction data but needs broader multicenter validation [40]. *TUBB3*, *MCL-1*, and pathway-based biomarkers remain exploratory and are derived from relatively small, single-center retrospective studies [44,45,50,52]. AI-enhanced transcriptomic tools such as GemPred and Pancreas-View [56,57], as well as ctDNA-based approaches [58,59], are conceptually appealing but lack prospective validation for regimen selection specifically. Importantly, the PASS-01 molecular correlative analyses, while representing the first randomized biomarker data in this setting, were derived from a phase II trial and were not statistically powered for definitive subgroup conclusions; the observed associations between transcriptomic subtype and regimen benefit should therefore be considered hypothesis-generating pending confirmation in larger studies [9]. Additionally, none of the biomarker studies reviewed in this manuscript included NALIRIFOX, which is now listed as an NCCN-preferred first-line regimen. Whether the biomarker-regimen associations described for mFOLFIRINOX and GnP extend to NALIRIFOX remains entirely unknown and represents a critical evidence gap.

Beyond the NALIRIFOX gap, none of the biomarker-regimen associations reviewed in this manuscript were tested in the context of *RAS*-targeted therapy. The ongoing phase 3 daraxonrasib trials evaluating *RAS* inhibition in both the first-line metastatic (NCT07491445) and adjuvant (NCT07252232) settings may render some of the current biomarker questions regarding FOLFIRINOX-versus-GnP selection less central if *RAS* inhibition becomes a first-line standard for the approximately 90% of PDAC patients with *KRAS* mutations [36,37]. Similarly, the elraglusib Phase 2 data, while promising, require Phase 3 confirmation before the associated biomarkers (*CXCL2*, *TRAIL*, cytokine panels) can be considered for clinical decision-making [61]. The POLAR trial, while demonstrating encouraging outcomes for pembrolizumab plus olaparib maintenance in HRD-positive metastatic PDAC, was a single-arm phase 2 study and requires randomized confirmation, which the ongoing SWOG S2001 trial is designed to provide [29]. Beyond these evidence-level limitations, several practical barriers currently limit the routine clinical implementation of biomarker-guided regimen selection in metastatic PDAC. Tissue availability remains a fundamental constraint. Most metastatic PDAC diagnoses rely on endoscopic ultrasound-guided fine-needle biopsies that frequently yield limited material, which must be prioritized among competing demands for histologic diagnosis, immunohistochemistry, and molecular profiling. RNA quality from FFPE specimens is often degraded, reducing the reliability of transcriptomic classifiers. Turnaround time for comprehensive molecular profiling typically ranges from two to four weeks, creating a clinical dilemma between initiating empirical chemotherapy and waiting for results that might inform regimen selection. Finally, cost and accessibility present significant barriers, as advanced profiling platforms such as whole-genome sequencing and RNA-seq remain largely confined to academic centers, and payer reimbursement for comprehensive molecular profiling in PDAC is inconsistent across healthcare systems.

Finally, no biomarker discussed in this review has been validated in a prospective, biomarker-stratified randomized trial that uses regimen selection as the primary endpoint. Until such trials are completed, all biomarker-guided recommendations should be considered hypothesis-generating rather than practice-defining.

## 10. Conclusions

Metastatic PDAC is beginning to move beyond purely empirical chemotherapy selection, but the field remains in transition. The recent head-to-head randomized trials have challenged the longstanding assumption of FOLFIRINOX superiority and demonstrated that GnP is at least equivalent, and possibly superior, in unselected populations [9,10]. This finding strengthens the rationale for biomarker-guided selection: if the regimens are roughly equivalent in unselected populations, identifying molecular subgroups that differentially benefit from one regimen becomes clinically essential. Among available biomarkers, HRD, defined by germline *BRCA1/2* or *PALB2* alterations and increasingly by broader genomic scar signatures, provides the strongest current rationale for selecting FOLFIRINOX or another platinum-based regimen [23,24,28]. *KRAS* mutation subtype analysis adds an emerging layer, with retrospective data suggesting that FOLFIRINOX may be preferred for the common G12D, G12V, and G12R variants [34]. However, conflicting data for the rare G12C subgroup highlight the need for prospective validation [34,35]. Transcriptomic subtype and *GATA6* expression are promising additional tools, though conflicting evidence between observational studies and the PASS-01 randomized trial tempers their current utility for regimen selection [9,21,22]. For GnP, hENT1 expression and *TUBB3* absence offer the most biologically grounded and clinically validated signals [40,41,44,45], while pathway-specific mutations such as *PI3K* and *KIT*, and *MCL-1* expression, provide hypothesis-generating support [50,52].

AI-enhanced transcriptomic classifiers such as GemPred and Pancreas-View represent promising near-term tools for enabling drug-specific regimen selection, as they generate predictions for individual chemotherapy components rather than broad prognostic categories [56,57]. ctDNA may complement tissue-based profiling by enabling non-invasive molecular characterization and adaptive treatment monitoring [58]. PDO pharmacotyping, as being evaluated in PASS-01, offers an additional functional approach to personalized regimen selection [9].

Whether the biomarker-regimen associations described in this review for mFOLFIRINOX and GnP extend to NALIRIFOX, now an NCCN-preferred first-line option, remains an important unanswered question and should be a priority for future translational research, as does the broader role of comprehensive profiling in identifying actionable alterations beyond chemotherapy selection. The most useful near-term clinical model is therefore an integrated one: combine tumor biology with patient fitness and treatment goals, recognize that predictive evidence remains strongest for HRD, and use broad molecular testing to identify opportunities for targeted therapy whenever present. Prospective validation through biomarker-stratified randomized trials remains required. Meanwhile, the emergence of pan-*RAS* inhibitors in phase 3 first-line trials could redefine the role of *KRAS* mutation status from a prognostic marker to a primary treatment-selection biomarker [36,37], and the POLAR trial has expanded the therapeutic implications of HRD testing beyond platinum selection [29]. These developments, together with the convergence of genomic, transcriptomic, and functional profiling approaches, suggest that a biologically informed treatment paradigm for metastatic PDAC is now both plausible and increasingly necessary.

## Figures and Tables

**Table 1:** Summary of Candidate Biomarkers for Regimen Selection in Metastatic PDAC.

Biomarker	Biomarker Classification	Regimen Signal	Key Finding	Evidence Level	Refs
Germline *BRCA1/2* or *PALB2*	Predictive (clinically actionable)	FOLFIRINOX	ORR 58% vs. 21%; PFS 10.1 vs. 6.9 mo (mutant vs. WT)	Retrospective cohort	[23]
HRD genomic scar signature	Exploratory predictive	FOLFIRINOX	HR 0.37; OS 12.8 vs. 4.5 mo (HRD+ on FFX vs. GnP)	Retrospective cohort	[24]
*BRCA2* pathogenic variant	Predictive (clinically actionable)	FOLFIRINOX	ORR 66.7% vs. 33.3% (FFX vs. GnP); OS comparable due to treatment sequencing (*n* = 4356)	Retrospective cohort	[30]
Classical subtype (PurIST)	Prognostic and exploratory predictive (conflicting data)	Conflicting	HR 0.67 favoring FFX in observational data; opposite in PASS-01 (OS 13.9 vs. 9.7 mo favoring GnP in classical)	Prospective RCT + Retrospective cohort	[9,22]
*GATA6*-high (IHC)	Prognostic and exploratory predictive	FOLFIRINOX	OS 24.9 vs. 10.8 mo (*GATA6*-high vs. low on FFX); no difference in untreated	Retrospective cohort	[21,33]
Basal-like subtype	Prognostic only	Neither	Poor prognosis regardless of regimen; strongly basal is independent predictor of worse outcomes (*n* = 8743)	Retrospective cohort	[16]
*KRAS* G12D/G12V/G12R	Prognostic; exploratory predictive for regimen; emerging targeted therapy biomarker	FOLFIRINOX	Longer TTNT and OS with FFX vs. GnP across most subtypes (*n* = 2433)	Retrospective cohort	[34]
*KRAS* G12C	Prognostic; targeted therapy selection (adagrasib, sotorasib)	Conflicting	One study: OS 15.7 vs. 8.0 mo favoring GnP; another: FFX associated with longest TTNT	Retrospective cohort	[34,35]
hENT1-high	Predictive (treatment-specific interaction demonstrated)	GnP	ORR 43% vs. 21%; OS 10.6 vs. 6.7 mo (high vs. low in GnP arm); no difference on FFX; interaction *p* = 0.002	Prospective observational	[40]
*TUBB3*-low	Predictive (treatment-specific for GnP)	GnP	DCR 100% vs. 64%; PFS 7.1 vs. 3.7 mo (TUBB3-negative vs. positive on GnP)	Retrospective cohort	[44]
*TUBB3*-low/hENT1-high combined	Predictive (treatment-specific for GnP)	GnP	OR 11.96 for disease control; HR 0.33 for PFS (combined score on GnP)	Retrospective cohort	[41]
*SMAD4* alteration	Prognostic; exploratory predictive (negative predictor for FOLFIRINOX; localized setting only)	GnP (negative predictor for FOLFIRINOX)	Metastatic progression 30.0% vs. 14.5% (*p* = 0.009) on FFX in *SMAD4*-altered; no difference on GnP	Retrospective cohort (localized setting)	[53,54]
*PI3K/KIT* pathway mutations	Exploratory predictive	GnP	*PI3K*: PFS 6.6 vs. 5.7 mo; *KIT:* PFS 10.3 vs. 6.2 mo (mutant vs. WT in GnP cohort); no difference on FFX	Retrospective cohort	[52]
*MCL-1* expression	Exploratory (prognostic vs. predictive uncertain; no comparator arm)	GnP (no FFX comparator)	OS 14.9 vs. 9.2 mo (*MCL-1* positive vs. negative on GnP; *n* = 38)	Retrospective cohort	[50]
*SPARC*	No signal (negative biomarker)	No signal	No association with OS, PFS, or ORR in MPACT exploratory analysis	Phase III exploratory	[49]

Abbreviations: FFX = FOLFIRINOX; GnP = gemcitabine/nab-paclitaxel; ORR = objective response rate; OS = overall survival; PFS = progression-free survival; DCR = disease control rate; HR = hazard ratio; OR = odds ratio; mo = months; WT = wild-type; IHC = immunohistochemistry; TTNT = time to next treatment. Gray shading is used for alternating rows to improve readability.

## Data Availability

No new data were created or analyzed in this study. Data sharing does not apply to this article.

## Abbreviations

The following abbreviations are used in this manuscript: AI—artificial intelligence; *ARID1A*—AT-rich interaction domain 1A; *ATM* ataxia-telangiectasia mutated; *BCL-2*—B-cell lymphoma 2; *BRAF*—v-Raf murine sarcoma viral oncogene homolog B; *BRCA1/2*—breast cancer gene 1/2; C-CAT—Center for Cancer Genomics and Advanced Therapeutics; *CDKN2A*—cyclin-dependent kinase inhibitor 2A; *CHEK2*—checkpoint kinase 2; CI—confidence interval; COMPASS—Canadian Oncology Molecular Profiling in Advanced Cancers Trial; ctDNA—circulating tumor DNA; ctDNA-RECIST—circulating tumor DNA response evaluation criteria in solid tumors; *CTLA4*—cytotoxic T-lymphocyte-associated protein 4; *CXCL2*—C-X-C motif chemokine ligand 2; DCR—disease control rate; DDR—DNA damage response; dMMR—deficient mismatch repair; ECOG—Eastern Cooperative Oncology Group; EMT—epithelial-to-mesenchymal transition; *FANCA*—Fanconi anemia complementation group A; FDA—Food and Drug Administration; FFPE—formalin-fixed paraffin-embedded; *FGFR2*—fibroblast growth factor receptor 2; FOLFIRINOX—fluorouracil—leucovorin—irinotecan—and oxaliplatin; *GATA6*—GATA-binding protein 6; GnP—gemcitabine plus nab-paclitaxel; *GSK-3β*—glycogen synthase kinase 3 beta; GTP—guanosine triphosphate; *hENT1*—human equilibrative nucleoside transporter 1; *HER2*—human epidermal growth factor receptor 2; HR—hazard ratio; HRD—homologous recombination deficiency; HRDsig—homologous recombination deficiency signature; HRR—homologous recombination repair; IHC—immunohistochemistry; JCOG—Japan Clinical Oncology Group; *KRAS*—Kirsten rat sarcoma viral oncogene homolog; LOH—loss of heterozygosity; LST—large-scale state transitions; *MCL-1*—myeloid cell leukemia 1; mFOLFIRINOX—modified FOLFIRINOX; MMR—mismatch repair; MSI-H—microsatellite instability-high; *MTAP*—methylthioadenosine phosphorylase; NALIRIFOX—liposomal irinotecan—fluorouracil—leucovorin—oxaliplatin; NCCN—National Comprehensive Cancer Network; NGS—next-generation sequencing; *NRG1*—neuregulin 1; *NTRK*—neurotrophic tyrosine receptor kinase; OR—odds ratio; ORR—objective response rate; OS—overall survival; *PALB2*—partner and localizer of BRCA2; PanIN—pancreatic intraepithelial neoplasia; PARP—poly(ADP-ribose) polymerase; PASS-01—Pancreatic Adenocarcinoma Signature Stratification for Treatment; PDAC—pancreatic ductal adenocarcinoma; *PD-1*—programmed cell death protein 1; *PD-L1*—programmed death-ligand 1; PDO—patient-derived organoid; PFS—progression-free survival; *PI3K*—phosphatidylinositol 3-kinase; PS—performance status; PurIST—Purity Independent Subtyping of Tumors; *RET*—rearranged during transfection; RNA-seq—RNA sequencing; *RNF43*—ring finger protein 43; *SMAD4*—SMAD family member 4; *SPARC*—secreted protein acidic and rich in cysteine; SWOG—Southwest Oncology Group; TAI—telomeric allelic imbalance; *TGFBR2*—transforming growth factor beta receptor 2; *TIM3*—T-cell immunoglobulin and mucin domain-containing protein 3; *TP53*—tumor protein p53; *TRAIL*—tumor necrosis factor-related apoptosis-inducing ligand; TTNT—time to next treatment; *TUBB3*—class III β-tubulin; *UGT1A1*—uridine diphosphate glucuronosyltransferase 1A1; WT—wild-type.
